# Scoping review of pharmacological and non-pharmacological management of agitation in post traumatic brain injury patients

**DOI:** 10.1186/s41016-026-00445-7

**Published:** 2026-08-11

**Authors:** Irrum Aneela, Priya Kadam, Chandrasekaran Kaliaperumal

**Affiliations:** 1 Rehabilitation Medicine, Astley Ainslie Hospital, Edinburgh, UK; 2 Department of Clinical Neurosciences, Royal Infirmary of Edinburgh, Edinburgh, UK

**Keywords:** Traumatic brain injury, TBI, Agitation, Management, Drug, Non-pharma

## Abstract

**Background:**

Traumatic brain injury (TBI) is a major cause of morbidity and mortality worldwide. Agitation is a common complication after moderate to severe TBI and managing it in the acute hospital setting can be challenging.

**Aim:**

Currently, there are no standardised interventions to manage agitation in TBI. This review aims to evaluate contemporary research over the past 10 years which addresses pharmacological and non-pharmacological strategies for managing agitation post-TBI.

**Method:**

A scoping review literature search was conducted using PRISMA guidelines. The protocol was pre-registered on PROSPERO (ID 1295903). The literature search used Mesh and Boolean keywords ‘traumatic brain injury and ‘agitation management’ in PubMed, and a free-text search of ‘traumatic brain injury and agitation management’ with ‘map term to subject heading’ in Embase (Ovid) and Cochrane, covering studies from 01 January 2015 to 09 March 2026. Findings were analysed and discussed between two reviewers.

**Results:**

A total of 65 studies were identified, and after careful review, 13 papers were selected from PubMed and Embase for our Scoping Review. Non-pharmacological strategies highlighted the importance of orienting the patient and the supportive role of family during the acute recovery period. Pharmacological strategies included found that the antipsychotic olanzapine was helpful in managing agitation in moderate to severe TBI. Valproic acid in a small male sample [1] and a separate study using dextromethorphan and quinidine [2] found that these medications were also helpful in managing agitation in TBI.

**Discussion and conclusion:**

Clinicians and health professionals reported that treating the patient holistically, with the medical, social, and psychiatric history was of benefit. Antipsychotic Olanzapine and Valproic acid may have their uses in the short-term, interim acute setting under supervision. Future research with larger sample sizes further investigating these medications would be useful, along with personalisation of medications to the patient’s medical history. Most of the studies included in the review are observational and reviews, rather than randomised controlled trials, with a lack of primary evidence which may be a limitation.

**Systematic review registration:**

PROSPERO CRD 1295903

**Supplementary Information:**

The online version contains supplementary material available at 10.1186/s41016-026-00445-7.

## Background

Traumatic brain injury (TBI) occurs when an external force causes damage to the brain, often through mechanisms such as falls or road traffic accidents [3]. TBI is a leading cause of disability [4], mortality, and morbidity worldwide [3]. In the USA, there were approximately 214,110 TBI-related hospitalizations in 2020 and 68,663 TBI-related deaths in 2023. These figures represent more than 586 TBI-related hospitalizations and 190 TBI-related deaths per day [5]. It is important to note that estimated figures do not include the many TBIs that are only treated in the accident and emergency department, primary care, urgent care, or those that go untreated [5, 6].

Notably, agitation has been reported in 20–41% of patients during the early stage of recovery in acute care units and up to 70% of patients in rehabilitation units [7].

Agitation following TBI comprises restlessness, disinhibition, impulsive behaviour, variable anger, irritability, and aggression. Formally, agitation is defined by the International Psychogeriatric Association as falling into one of four criteria: (A) behaviours attributed to cognitive impairment (B) behaviours present for 2 weeks or more which are different from the individuals' normal behaviour including one of (i) verbal and/or (ii) physical aggression or (iii) excessive motor activity, behaviour causing distress or significant impairment to social or daily functioning or D) behaviours which are not attributed to a psychiatric state or medication [8].

It affects 11 to 70% of patients after TBI admitted to acute care wards and typically persists for 1 to 14 days, sometimes longer before they can be discharged [9].

Possible pre-morbid risk factors for agitation include a history of substance use, mood and personality disorders, criminal record, dysregulated aggression, and antisocial behaviour [9]. Additionally, a longer duration of lost consciousness and the presence of co-morbid medical conditions are also factors that may lead to agitation post-TBI [9].

Potential risk factors for post-TBI agitation include infection, poor sleep, frontal lobe injury, environmental overstimulation, pain, and age [10].

There is a strong relationship between agitated behaviour and frontal lobe injury, which leads to disinhibition and lack of awareness of the associated outcome [11]. Agitation also prolongs the duration of post-traumatic amnesia and causes impairment of motor and cognitive functioning [12].

Agitation may lead to injury, as patients can become disorientated and risk falling due to wandering. They may forget to ask nurses for help before mobilising. This can delay rehabilitation if injury occurs and requires additional management plans. Longer inpatient stays may worsen disorientation and agitation, making management harder. Agitated patients may need restraints, which prolong hospitalisation and disrupt independence [10, 12].

The change in behaviour may also affect their relationships with friends and family, thereby risking damage to their social life [7].

Agitated behaviour negatively impacts staff wellbeing and can lead to burnout. These patients increase staff workload by wandering due to a lack of safety awareness. They require constant supervision and raise the risk of falls [12]. Therefore, they are at risk of further injury, including worsening brain injuries and fractures, which can increase the severity of TBI and delay rehabilitation outcomes.

The impact of agitation post-TBI can be seen to affect patients, families, and staff, and using evidence-based strategies to manage agitation is essential. This review will examine the latest evidence-based strategies to manage this challenging post-TBI presentation.

## Method

### Search description and rationale

Database searches were conducted in PubMed, Cochrane Reviews, and Embase (Ovid).

The search terms were developed with the assistance of a clinical librarian at Birmingham University Hospitals Foundation Trust (UK). The searches took place between 01 January 2015 and 09 March 2026. A combination of medical search terms was used, and has been outlined below:

- 1) PubMed Mesh Terms with Boolean operator: Traumatic Brain Injury AND agitation management’
- 2) Embase (Ovid): An advanced free text search for ‘Traumatic Brain Injury and agitation management’. Note: ‘Map to subject headings’ box (MeSH) was ticked.
- 3) Cochrane used a free text search for ‘Traumatic Brain Injury and agitation management’. Non-advanced search term

### Study selection and eligibility

The 27-point Prisma (2020) checklist was used to ensure standardised reporting was maintained and that the research methodology was transparent (see Supplementary Material).

Duplicates were removed in the first stage of title screening and again at abstract screening.

The titles were screened for relevance to the search topic: ‘traumatic brain injury and agitation management’.

#### Included

Papers which were published between 2015 and 2025 and addressed the topic of management of agitation and traumatic brain injury for a contemporary search of the literature and most up-to-date research. Once the topic was satisfied, the following types of papers were included: Systematic reviews, Descriptive analysis, International Survey, Randomised medicine-controlled trial, Randomised crossover trial design, Narrative Review, Clinical Practical Guidelines, Surveys, Interviews and conference abstracts were also included.

#### Excluded

Unrelated research which did not focus on the search theme (described as an ‘irrelevant topic’), letters, research with no abstracts, paediatric studies, and animal studies.

#### The screening process

The search results were replicated, and the results were discussed between two raters, who worked on separate sites. The clinical librarian and their team of two others aided were also able to replicate the searches on the three databases.

The papers which were included in the review were read in full by both reviewers and included in the results section. Scoring sheets were used for screening each study, where each rater would tick the appropriate reason for inclusion or exclusion of a research study on screening title and/or abstract based on the inclusion and exclusion criteria outlined above (please see Supplementary Material for the scoring sheets).

The preliminary search was registered on PROSPERO.

### Identification of bias

A few of the papers contained qualitative data and the CASP (Critical Appraisal Skills Programme) checklist [13] was used to assess methodology and allowed reviewers to validate the study results. It was decided that the CASP tool would suit the number of qualitative studies included in the review, rather than the Joanne Briggs [5].

### Data preparation, synthesis, and presentation

Initially, during the screening process, paper scoring sheets were used for screening each study. Results were then tabulated on the computer in Microsoft word for comparison and to check consistency between raters.

Data synthesis was carried out with discussion after reading each article and visually assessing between both reviewers, where the 65 articles being screened, and the papers included were compared and discussed. Disputes around replication of data, inclusion of paediatric studies were settled by discussion and consensus was reached.

Data were prepared in Microsoft Word and then imported to Microsoft Excel, before being converted into graphical and table format.

## Results

**Fig. 1:**
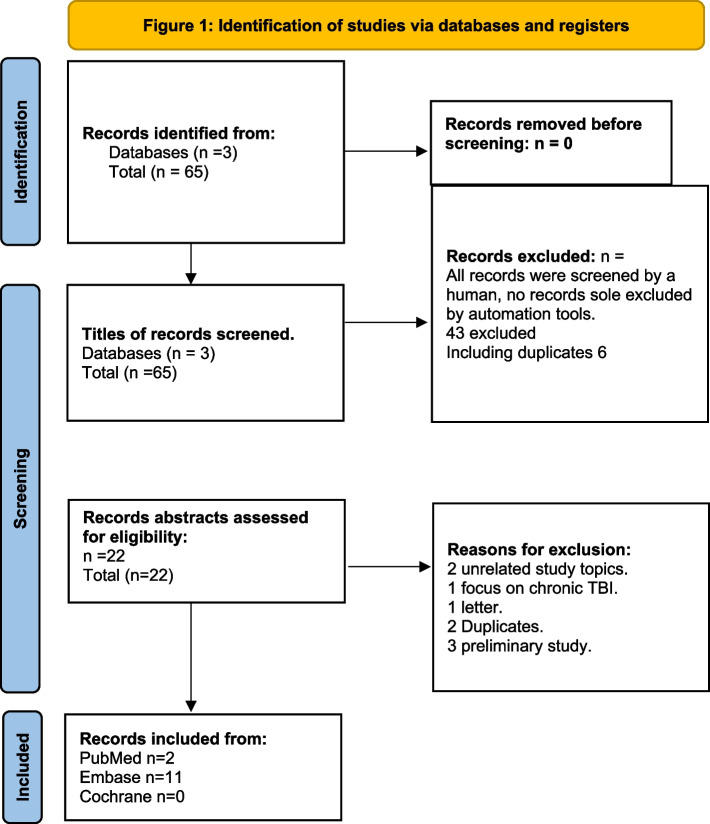
Identification of studies via databases and registers

**Fig. 2:**
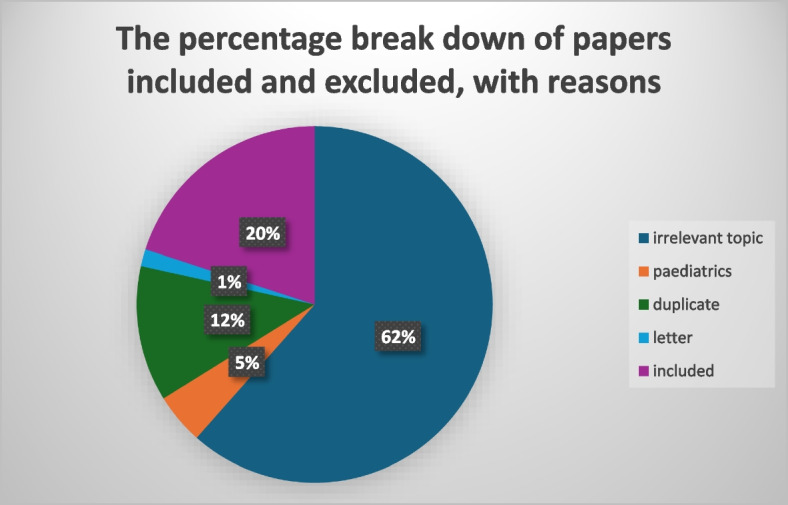
Above: Reasons for the papers excluded in the review, by percentage, compared with the percentage of papers included in the review, as a proportion of the total. Overall papers were excluded for the following reasons: 65% (40 papers) were irrelevant topics, 12% (8 papers) were duplicates, 5% (3 papers were paediatric), 1% (1 paper) was a letter and 20% of the total 65 papers screened [14]

**Fig. 3:**
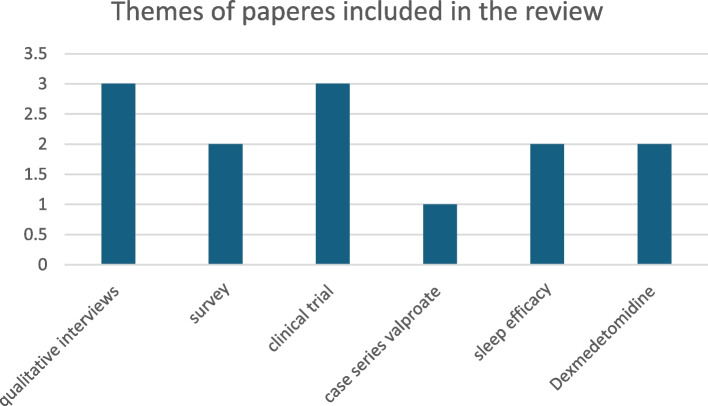
Graphical representation of the numbers of different types of studies included in the review

The database search identified 65 records, of which 13 were selected for full text review (diagram 1). The search terms used found 8 duplicates (including papers which were identified in the search results along with a conference paper with the same results) and topics which were unrelated, such as those addressing chronic TBI (please see Table A1 in the Supplementary Material, which details reasons for exclusion) (Figs. 1, 2 and 3).

Studies were international. Including one qualitative study which alone conducted interviews with 33 clinicians from 16 countries.

The following paragraphs group the findings of the papers included in this review into themes. The definition of agitation was commonly described as aggression and restlessness [15] but was not consistently defined across papers (Tables 1 and 2).

**Table 1:** Breakdown of reasons for the exclusion of papers and the number of papers in each category. The number of included studies has been provided for a comparison

Reason for exclusion	Number of papers
Irrelevant	40
Paediatrics	3
Duplicate	8
Letter	1
Comparison: Included	13

**Table 2:** A further breakdown of reasons for exclusion for the 40% of papers in the category of ‘irrelevant topics. Nineteen papers had spoken about TBI but not focused on the management of agitation

Breakdown of irrelevant topic	Number of papers
Medical complications of TBI	1
Delirium	1
Paroxysmal hyperactivity	1
Other drug treatment for TBI	7
Sedation	4
Preliminary/incomplete study	4
Agitation but not TBI	2
Non-drug TBI treatments not for agitation	1
Drug safety in TBI	1
TBI course with age	1
Non-drug treatment for TBI	2
Hormone investigation post TBI	1
Qualitative	1
Immersive reality	1
Other drug treatment for TBI	1
Chronic management	3
Unrelated intervention	1
Stimulant therapy in TBI	1
Sleep and assessment	1
Pupils and TBI	1
Amnesia	1
Paroxysmal	1
Behavioural problems in TBI	1
Cognitive deficit TBI	1
Total	40

### Current strategies of health professionals to manage agitation

A qualitative survey of 33 clinicians from 16 countries with experience working with agitated post-TBI patients found that in practice the following strategies reduced agitation: proactively removing potential sources of agitation (patient-related and environmental) and using consistent, simple behavioural management reduced agitation [16].

ICU nurses participated in semi-structured qualitative interviews, which were analysed thematically. Five main themes which worked for managing agitation emerged: (1) symptoms differ by patient profile and time since awakening; (2) agitation management approaches vary due to different concerns; (3) nurses use various strategies to manage agitation; (4) contextual factors influence management; and (5) there are opportunities to improve the integrated care model with a holistic perspective [17]. These results were not absolute and did not necessarily specify the type of technique or frequency which these techniques were used and for what severity of TBI.

### Environmental management of agitation

A cross-sectional anonymous online survey examined how 331 clinicians managed agitation during early recovery from TBI. The survey found that the most frequently used non-pharmacological strategies were reducing noise levels (80% of respondents) and managing patients in single rooms (81%) [15]. These findings are clinician opinion rather than evidenced based methods for techniques to reduce agitation. The strategies from the online survey may be useful but they are not yet supported by comparative trials.

Additionally, observational studies and conference extracts found that many clinicians considered sleep essential for recovery, noting that low sleep efficacy was associated with greater agitation [18].

A retrospective observational study found that, on day 1 post moderate to severe traumatic brain injury, patients with agitation had lower sleep efficacy. Since agitation negatively affects rehabilitation outcomes, minimising disruptive sleep patterns post-TBI is important. Sleep hygiene strategies may help reduce agitation post-TBI [4].

### Supportive management from family

**Fig. 4:**
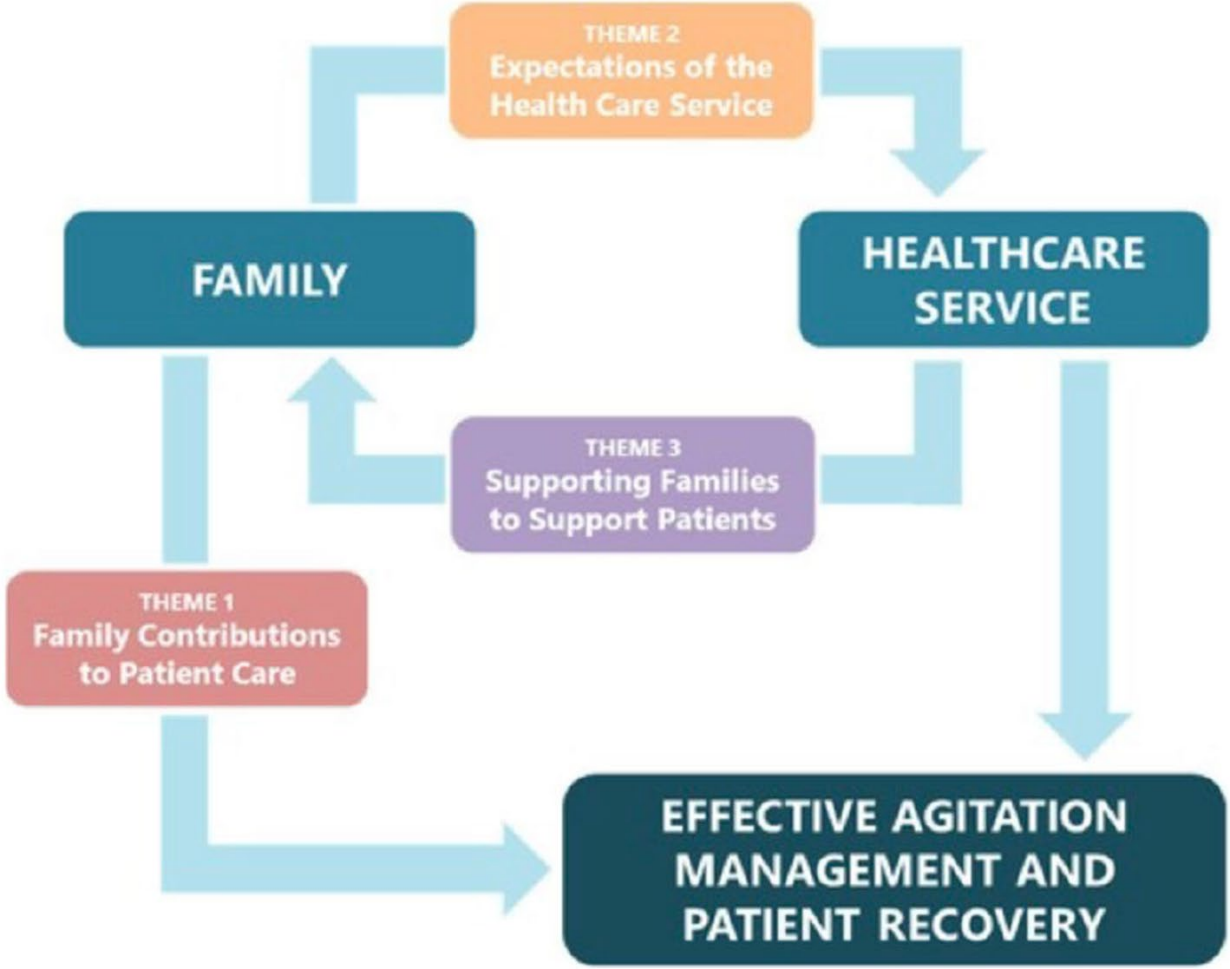
Above detailing the themes which emerged from the thematic analysis of 20 qualitative semi-structured interviews of families exploring their feelings surrounding holistic needs [19]. The themes identified, which families considered important (1) family contributions to patient care; (2) expectations of the health care service; (3) supporting families to support patients

Overall, families felt that their involvement during the early stages of traumatic brain injury recovery was beneficial for reducing the patient's agitation [12, 19]. Comforting strategies such as holding the patient’s hand, stroking the patient’s head, and supporting their loved one with physical touch helped the patient feel safe and secure [19]. Using similar methods suggested by the clinicians in the qualitative study [16], addressing the patient's environment by adding familiar features and belongings, and creating a place of calm [19]. In addition, the relationship between the care team and the family is important, as 20 qualitative semi-structured interviews explored families’ feelings about receiving sufficient information and having their support needs addressed to provide holistic care for the patient [19] (Fig. 4).

### Medical management of agitation

Canadian intensivist doctors addressed both the medical and environmental management of patients with agitation following TBI. In a 42-item survey of 219 physicians working in 18 ICU-level 1 trauma centres, 72% believed that alpha–2–adrenergic agonists were effective in managing agitation [14].

In a double-blind, randomised clinical trial researching the effectiveness of antipsychotic olanzapine vs placebo (11 participants, 5 oral olanzapine, and 6 placebo) for treatment of agitation in post-traumatic amnesia following TBI, it was found that olanzapine was effective at managing agitation overall in the post-traumatic amnesia phase, but not otherwise for the remaining duration of the TBI agitation [20]. The effective olanzapine dose ranged from 5 to 20 mg, and it was also highlighted that the drug may cause confusion in the agitated patient [20].

Short-term use of valproic acid (VPA) was useful for managing agitation in TBI in a small case series of 3 male patients, with doses ranging from 250 mg to 1 g daily. Their improvement was assessed using the Richmond Agitation-Sedation Scale (RASS). The VPA was used for 7 to 20 days prior to discontinuation, and liver function was monitored [1].

In a systematic review directly addressing pharmacological interventions for agitation in TBI, amantadine and olanzapine were more specifically found to reduce aggression, whereas valproic acid may reduce agitation [21]. However, the quality of the studies was found to be weak, as 11 of the 170 studies in the systematic review did not address agitation as the presenting symptom, and another 11 measured agitations as an outcome variable [21].

In a first reported case study of a conference abstract format, dextromethorphan, and quinidine in patients with post-traumatic brain injury were found to be beneficial and reduce agitation. However, adverse effects of cognitive impairment and QT prolongation were noted on ECG [22].

### Sedation as management for agitation

Dexmedetomidine, a sedating and analgesic agent that stimulates alpha-2 adrenergic receptors in the locus coeruleus region of the spinal cord, is another agent that has been trialled for agitation in TBI.

One paper that examined the use of Dexmedetomidine in traumatic brain injury found that within 72 h of dexmedetomidine initiation, the proportion of agitated patients had decreased from 100 to 63% [23].

The paper found that bradycardia and hypotension were common side effects in patients taking dexmedetomidine [23], in 76% and 54% of patients, respectively [23].

### Randomised controlled trials

The Cochrane review trials were not included in the paper because they were either irrelevant, duplicates of previous papers, or preliminary or future studies with no results. The duplicate paper was one that already included ‘Use of olanzapine to treat agitation in traumatic brain injury: a series of N of One trial.

### The perspective of clinicians in the management of agitation in TBI

Many clinicians reported dissatisfaction with their current agitation management guidelines and have insufficient training [15]. There is currently no consensus on the most efficacious and safest strategy to treat agitation post-TBI [10].

### Biases and criticisms of study methodology

In qualitative study exploring the needs of the family from services, the age of the patients who had sustained TBI varied, from age 18 to 86 [19]. Therefore, some of the needs of that patient may vary purely from an age perspective and may not be associated with TBI. The factor of age shaping health needs and current comorbidities was not focused on, and these may have contributed to agitated behaviours. In the same study, there was a very broad range of average time since injury, reported 296.50 days (SD = 177.80, range = 54–775 days) [19]. Similarly, the needs of the patient and the families may vary as they are in different stages of their recovery, which may make it more difficult to compare the needs of families and/or patients.

Another study noted in their limitations that the objective measure of agitation was not consistently measured, and therefore this creates an unreliable finding which may not be useful for clinicians hoping to use the results from this study in practice.

To note that there was only one original RCT included in the review. Results of a double-blind, randomised, placebo-controlled trial investigating the efficacy of olanzapine for agitation management, reported that there were limitations to the study, as olanzapine is also used as rescue medication. A decision was made due to safety concerns to favour the use of olanzapine rather than any other confounding antipsychotic medication to the trial design. There were reported ‘blurred lines between olanzapine and placebo allocation' as a result, which may affect a bias of patient allocation and/or treatment during the trial [20].

CASP checklist for qualitative studies.

## Discussion

Overall, this review found that there was a mixture of both pharmacological and non-pharmacological methods of managing agitation after traumatic brain injury, with some strategies reported by clinicians without quantifying evidence of success and others outlined in randomised controlled trials.

Not all the studies included in the review addressed agitation as their primary or presenting symptom; this may have created a reporting bias as researchers likely focused on different behavioural symptoms primarily [21]. The broad definition of agitation may also be a confounding issue, as some participants may fall into one or more of the criteria for agitation [8] and therefore different types of agitation may be managed well using different strategies.

Populations were of different ages and therefore recovery, past medical history and severity of TBI may have responded differently to different agitation management strategies and there is a difficult comparator.

Nonpharmacological methods such as reducing noise, giving patients their own room, and removing sources of agitation [4, 15] were considered beneficial by health professionals to reduce agitation and the large body of respondents gained consensus. Putting together these practices into guidelines may be the next step. Challenges may include creating universal non-pharmacological guidelines, as language and non-verbal behaviour may be observed and viewed differently across different countries. Therefore, describing ‘agitated behaviour’ and the desired outcome of ‘reduction in agitation’ may vary.

In contrast, pharmacological studies were often small clinical trials in the intensive care unit [1, 20, 21]. By comparison, the studies with larger sample sizes were often surveys of intensivists and their perspectives on 1) the current treatment of agitation in TBI and 2) which management strategies currently work well, rather than experimental studies [14, 15].

Smaller sample sizes (2, 1, and 16) may require caution when interpreting the results of a single study. However, there is strength when examining more than one study, by independent researchers who have come to the same or very similar conclusions.

For example, the antipsychotic olanzapine was found to be effective overall in reducing agitation post-TBI in one primary trial with a small sample size [20] and as an outcome of a systematic review [21].

More specifically, limitations of one of the studies looking at the use of dexmedetomidine as a cooperative sedation agent to reduce agitation post TBI were that there were small sample sizes [23], and the agent may be unsuitable for individuals with TBI who have existing bradycardia and/or hypotension [23]. A risk-based analysis to weigh up the use of Dexmedetomidine would be needed with senior and ICU involvement if the agent were to be used in patients who had bradycardia and/or hypotension. The authors advise caution when interpreting the results of the same study, as the frequency of agitation assessment (RASS) varied, and there was no control group to compare results [23].

The RASS agitation rating scale was commonly used as an objective screening tool to measure agitation pre and post drug dose in some of the clinical trials. Researchers in one paper highlighted that the RASS had been used in patient assessment [24] but reported that the patients were not always scored and therefore the reliability of results may be reduced, as there was no objective comparison for agitation pre and post drug dose. This acted as a reminder that standardised reporting outcomes were essential to validate results.

Families felt that being informed about the short term and long-term patient recovery was essential. In addition, being kept informed of the current plan for the patient and their needs, families may feel empowered to assist with their loved one's recovery and engage, which has been found to be beneficial [19].

The quality of the studies also varied from high quality RCT to observational and conference abstracts. The level of evidence varies from the primary to the protocol-driven studies which were proposed, and although were included in the scoping review by the search terms, the evidence needs to be considered separately from a hypothesis.

Future research may recruit larger sample sizes for clinical studies and may not necessarily be restricted to the intensive care unit but also neurosurgery and neurology units. Recruiting participants of a similar age and/or duration from TBI may provide more stratified guidelines for the types of patients who may benefit from olanzapine for the treatment of agitation. More specific research could evaluate the safety of dosing antipsychotics in patients with existing psychiatric histories.

## Conclusion

Overall, studies have highlighted the need for clinical training in managing agitation in TBI. Both non-pharmacological methods, such as reducing noise, orienting the patient, and supporting the family, have been reported by clinicians as strategies which they use to manage agitation post-TBI; however the reliability and evidence are lacking and not quantifiable being more opinion-based.

In addition, pharmacological methods such as olanzapine and valproic acid may have their uses in the short-term, interim acute setting under supervision; however the findings come from only a few studies. Further clinical trials to replicate these findings may be needed to facilitate guideline formation for managing agitation in TBI, along with larger sample sizes of participants.

## Supplementary Information

Supplementary Material 1.

## Data Availability

No datasets were generated or analysed during the current study.

## Abbreviations

TBI: Traumatic brain injury
CASP: Critical Appraisal Skills Programme
VPA: Valproic acid
RASS: Richmond Agitation-Sedation Scale
