# Supplementary material for: Scoping review of pharmacological and non-pharmacological management of agitation in post traumatic brain injury patients
Source: Chin Neurosurg J. 2026 Aug 11;12:25. doi: 10.1186/s41016-026-00445-7 (PMC13459245; doi:10.1186/s41016-026-00445-7)
Supplement: Supplementary file 1 [file 41016_2026_445_MOESM1_ESM.docx]

Appendices

A) Supplementary Table 1 – exclusion criteria

| Study | Title included Y/N | Abstract  Y/N | Reason for exclusion |
| --- | --- | --- | --- |
| Study 1 | N | N | Study in Paediatric (wrong population) |
| Study 2 | N | N | Excluded at title screening irrelevant topic |
| Study 3 | Y | N | Excluded at abstract screening irrelevant topic -Intervention not related to our study topic |
| Study 4 | Y | N | Excluded at abstract screening irrelevant topic |
| Study 5 | Y | N | Letter |
| Study 6 | Y | Y | Included in the review |
| Study 7 | Y | Y | Included in the review |
| Study 8 | N | N | Excluded at title screening focus on chronic management |
| Study9 | N | N | Study in Paediatric (wrong population) |
| Study10 | N | N | Excluded at title screening irrelevant topic |
| Study 11 | N | N | Excluded at title screening irrelevant topic, Focus on chronic management |
| Study12 | N | N | Excluded at title screening irrelevant topic - |
| Study13 | N | N | Study in Paediatric (wrong population) |
| Study14 | N | N | Excluded at title screening irrelevant topic |
| Study15 | N | N | Excluded at title screening irrelevant topic |
| Study16 | N | N | Excluded at title screening irrelevant topic |
| Study17 | Y | N | Excluded at abstract screening irrelevant topic -Focus on chronic TBI outcomes |
| Study18 | N | N | Excluded at title screening – irrelevant topic.  Sedation in brain injury |
| Study19 | Y | N | Excluded at abstract screening due to preliminary incomplete study |
| Study20 | N | N | Excluded at title screening- duplicate |
| Study21 | N | N | Excluded at title screening- irrelevant topic: tranexamic acid in TBI |
| Study 22 | N | N | Excluded at title screening- irrelevant topic.  Dexmedetomidine in neonate |
| Study23 | Y | N | Excluded at abstract screening due to preliminary incomplete study |
| Study24 | Y | N | Excluded at abstract screening due to preliminary incomplete study |
| Study25 | N | N | Excluded at title screening irrelevant topic.  Propranolol for TBI |
| Study26 | N | N | Excluded at title screening irrelevant topic.  Safety of dexmedetomidine in TBI |
| Study27 | N | N | Excluded at title screening- duplicate |
| Study28 | N | N | Excluded at title screening irrelevant topic.  Inpatient TBI course with age |
| Study29 | N | N | Excluded at title screening irrelevant topic.  Cerebral deoxygenation in TBI |
| Study30 | N | N | Excluded at title screening irrelevant topic.  Abnormal cortisol after TBI |
| Study31 | N | N | Excluded at title screening irrelevant topic.  Sedation in TBI |
| Study32 | N | N | Excluded at title screening duplicate |
| Study 33 | N | N | Excluded at title screening irrelevant topic.  Economic activity in TBI |
| Study34 | N | N | Excluded at title screening irrelevant topic.  Sertraline in TBI |
| Study35 | N | N | Excluded at title screening irrelevant topic.  Dexmedetomidine on periop TBI |
| Study36 | N | N | Excluded at title screening irrelevant topic.  Sedation in TBI |
| Study37 | N | N | Excluded at title screening irrelevant topic.  Medical complications of TBI |
| Study38 | N | N | Excluded at title screening irrelevant topic.  Valproate reduces delirium |
| Study39 | N | N | Excluded at title screening irrelevant topic.  Sedation in TBI |
| Study40 | N | N | Excluded at title screening irrelevant topic.  Risperidone for irritability and aggression after head injury |
| Study41 | N | N | Excluded at title screening irrelevant topic immersive reality |
| Study42 | N | N | Excluded at title screening irrelevant topic. |
| Study43 | N | N | Excluded at title screening irrelevant topic.  Experiences and attitudes towards TBI ICU patients |
| Study 44 | Y | Y | Included in the review. |
| Study45 | Y | Y | Included in the review. |
| Study46 | N | N | Excluded at title screening irrelevant topic Hypothermia |
| Study47 | Y | N | Duplicate – excluded at level of abstract screen. Same results but different study |
| Study48 | Y | Y | Included in the review. |
| Study49 | Y | Y | Included in the review. |
| Study50 | Y | Y | Included in the review. |
| Study51 | N | N | Excluded as it is a duplicate the conference abstract for paper2 |
| Study52 | N | N | Excluded as it is a duplicate the conference abstract for paper 7 |
| Study53 | N | N | Excluded at title screening as it is a duplicate of 7 and 9 |
| Study54 | Y | N | Excluded at the abstract level as this is a conference duplicate of 5 |
| Study 55 | N | N | Excluded at title screening irrelevant topic.  Paroxysmal sympathetic hyperactivity |
| Study56 | N | N | Excluded at title screening irrelevant topic.  Neuroprotective nursing care in TBI |
| Study57 | N | N | Excluded at title screening preliminary study |
| Study58 | Y | Y | Included in the review. |
| Study59 | Y | Y | Included in the review. |
| Study60 | N | N | Excluded at title screening irrelevant topic.  MDT agitation |
| Study61 | Y | Y | Included in the review. |
| Study62 | N | N | Excluded at title screening irrelevant topic.  Management of general agitation |
| Study63 | Y | Y | Included in the review. |
| Study64 | Y | Y | Included in the review. |
| Study65 | Y | Y | Included in the review. |

B) Appendices of Publications included in the review.

Title Experiences and perceptions towards agitated behaviours in traumatic brain injury intensive care unit patients: Understanding various management practices of agitation through qualitative interviews with nurses.
Authors: Saavedra-Mitjans M., David P.-M., Arbour C.A., Perreault M.M., Roux M., Frenette A.J., Khwaja K., Bernard F.B.,
Williamson D.R.
Canadian Journal of Anaesthesia. Conference: Critical Care Canada Forum 2024. Toronto, ON Canada. 72(Supplement 1) (pp
81-83), 2025. Date of Publication: 01 Jun 2025.
AN: 650224773

Title Managing agitation during early recovery following traumatic brain injury: qualitative interviews with clinicians.
Authors Carrier S.L., Ponsford J., McKay A.
Disability and rehabilitation. 45(22) (pp 3686-3695), 2023. Date of Publication: 01 Nov 2023.
AN: 639359505

Title Experiences and attitudes towards agitated behaviours in TBI ICU patients (EXSTATIC): understanding various management practices through qualitative interviews with nurses.
Authors Saavedra-Mitjans M., David P.-M., Arbour C., Perreault M.M., Roux M., Frenette A.J., Khwaja K., Bernard F., Williamson D.R. Brain Injury. 39(7) (pp 598-609), 2025. Date of Publication: 2025.
AN: 2033042576

Title Physicians' beliefs and perceived importance of traumatic brain injury-associated agitation in critically ill patients: a survey of Canadian intensivists. Croyances et importance percue par les medecins de l'agitation associee aux traumatismes craniens chez la patientele gravement malade: un sondage realise aupres d'intensivistes au Canada
Authors Saavedra-Mitjans M., Frenette A.J., McCredie V.A., Burry L., Arbour C., Mehta S., Charbonney E., Wang H.T., Albert M.,
Bernard F., Williamson D.
Canadian Journal of Anesthesia. 71(2) (pp 264-273), 2024. Date of Publication: 01 Feb 2024.
AN: 2027274752

Title Use of Olanzapine to Treat Agitation in Traumatic Brain Injury: A Series of N-of-One Trials.
Authors Phyland R.K., McKay A., Olver J., Walterfang M., Hopwood M., Ponsford M., Ponsford J.L.
Journal of Neurotrauma. 40(1-2) (pp 33-51), 2023. Date of Publication: 01 Jan 2023.
AN: 2022494038

Title Managing agitation during early recovery in adults with traumatic brain injury: An international survey.
Authors Carrier S.L., Hicks A.J., Ponsford J., McKay A.
Annals of Physical and Rehabilitation Medicine. 64(5) (no pagination), 2021. Article Number: 101532. Date of
Publication: 01 Sep 2021.AN: 2013580188

Title A case series examining the use of valproic acid for agitation management after a traumatic brain injury.
Authors Diaz-Segarra N., McKay O., Yonclas P.
PM and R. Conference: 2020 Annual Assembly of the American Academy of Physical Medicine and Rehabilitation. Virtual. 12(SUPPL 1) (pp S111), 2020. Date of Publication: 2020.
AN: 634147323

Title Pharmacological interventions for agitation in traumatic brain injury: A systematic review.
Authors Williamson D., Frenette A.J., Perreault M., Burry L., Charbonney E., Lamontagne F., Potvin M.J., Giguere J.F., Mehta S., Bernard F.
Critical Care. Conference: 38th International Symposium on Intensive Care and Emergency Medicine, ISICEM 2018. Brussels Belgium. 22(Supplement 1) (no pagination), 2018. Date of Publication: 2018.
AN: 621461410

Title Analysis of sleep efficiency and agitation management in acute traumatic brain injury.
Authors Makley M., Weintraub A., Schneider H., Gerber D., Newman J., Philippus A., Biggs-Arnold J. Journal of Head Trauma Rehabilitation. Conference: 13th Annual Conference on Brain Injury of the North American BrainInjury Society's, NABIS 2016. Tampa, FL United States. 32(6) (pp E84), 2017. Date of Publication: 01 Nov 2017.
AN: 622461680

Title Pharmacological interventions for agitation in patients with traumatic brain injury: Protocol for a systematic review and meta-analysis.
Authors Williamson D.R., Frenette A.J., Burry L., Perreault M.M., Charbonney E., Lamontagne F., Potvin M.-J., Giguere J.-F., Mehta S., Bernard F. Systematic Reviews. 5(1) (no pagination), 2016. Article Number: 193. Date of Publication: 17 Nov 2016. AN: 613241033

Title Dextromethorphan and quinidine for treating post-traumatic brain injury agitation: A case series. Siu G., Vongviphut D. PM, and R. Conference: 2016 Annual Assembly of the American Academy of Physical Medicine and Rehabilitation, AAPM and R 2016. New Orleans, LA United States. 8(9 Supplement) (pp S247), 2016. Date of Publication: 01 Sep 2016.
AN: 612984565

Title: Safety of Dexmedetomidine for the Control of Agitation in Critically Ill Traumatic Brain Injury Patients: A Descriptive Study. Authors: V. Bilodeau, M. Saavedra‑Mutans, A.J. Frenette, L. Burry, M. Albert, F. Bernard, D.R. Williamson Journa**l:** *Journal of Clinical Pharmacy and Therapeutics* Date of Publication: 19 February 2021 (Volume 46, Issue 4, Pages 1020–1026) DOI: 10.1111/jcpt.13389 PMID: 33606290 Accession.AN: 080340357

Draganich, C., Gerber, D., Monden, K. R., Newman, J., Weintraub, A., Biggs, J., … Makley, M. (2019). Disrupted sleep predicts next day agitation following moderate to severe brain injury. *Brain Injury*, *33*(9), 1194–1199. <https://doi.org/10.1080/02699052.2019.1631484>

C) RASS Scoring system.

**Richmond Agitation and Sedation Scale (RASS)**

Found online at Physoiopedia: <https://www.physio-pedia.com/Richmond_Agitation-Sedation_Scale_(RASS)> (accessed 08 March 2026).

D) Screening scoring sheet

Scoring sheets for screening – one per study

| Criteria | Title level accepted Y/N | Abstract level accepted Y/N | Included in review Y/N |
| --- | --- | --- | --- |
| Inclusion criteria |  |  |  |
| Essential:  Relevant topic- TBI and agitation management |  |  |  |
| Published between 2015-2025 |  |  |  |
| Systematic review |  |  |  |
| Survey |  |  |  |
| Descriptive analysis |  |  |  |
| Interviews |  |  |  |
| International surveys |  |  |  |
| Randomised medicine-and randomised controlled trial |  |  |  |
| Narrative review |  |  |  |
| Clinical practice guidelines |  |  |  |
| Conference Abstracts |  |  |  |
| Randomised crossover trial |  |  |  |
| Exclusion criteria |  |  |  |
| Irrelevant topic |  |  |  |
| Letters |  |  |  |
| Research with no abstract |  |  |  |
| Paediatric studies |  |  |  |
| Animal studies |  |  |  |
| Descriptions of future research |  |  |  |
| Preliminary studies |  |  |  |
|  |  |  |  |

Supplementary table 4: Quality and Publication bias

| Study | Design and quantification tool | Limitation and /or data quality |
| --- | --- | --- |
| Managing agitation during early recovery in adults with traumatic  brain injury: An international survey | Survey based on the findings of literature review for clinicians. | Online survey looks at the opinion and types of strategies which clinicians use, rather than the primary evidence for efficacy. |
| Effectiveness of Non‐Pharmacological Interventions for Agitation  during Post‐Traumatic Amnesia following Traumatic Brain Injury:  A Systematic Review | Systematic review | absence of a control group, a lack of formal-  ised agitation measurement and inconsistent concomitant use of pharmacology. Interventions involving music therapy had  the highest level of evidence, although study quality was generally low to moderate. |
| Pharmacological interventions for agitation  in patients with traumatic brain injury:  protocol for a systematic review and meta-  analysis | Meta analysis | randomized controlled, quasi-experimental, and  Observational studies with control groups. The population of interest is all patients, including children and adults who have suffered a TBI.  A mixture of high quality and observational studies |
| Experiences and attitudes towards  Agitated behaviours in Traumatic brain  injury in the Intensive Care unit  patients (EXSTATIC): a protocol for an  interprofessional mixed-method study | Prospective study | Low impact as the results of the study is not outlined in this |
| Safety of dexmedetomidine for the control of agitation in  critically ill traumatic brain injury patients: a descriptive study | Descriptive drug trial  41 patients in whom dexmedetomidine was initiated to reduce agitation  The proportion of  patients with at least one episode of agitation decreased from 100% on day 0, to 88%,  69% and 63% on days 1, 2 and 3 of dexmedetomidine, respectively. The decrease was  statistically significant difference between days 0 and 2 as well as between days 0 and  3. Concomitant use of propofol and benzodiazepines also decreased over the course  of dexmedetomidine treatment. | Moderate level impact  Concomitant use of propofol and benzo may make results confounding or limited too patients who are also taking these medications in acute settings |
| Disrupted sleep predicts next day agitation  following moderate to severe brain injury | observational study of a convenience sample of 65 individuals  admitted to TBI inpatient rehabilitation | Low impact due to observational study |
| A descriptive analysis of pharmacological management of aggression and/or agitation in patients with traumatic brain injury in a Southwest Virginia inpatient population | Niche population  Descriptive analysis of observed effects of benzodiazepines /anti psychotics | Low to moderate impact as be observed study |
| Enhancing management of agitation after traumatic brain injury: Psychiatric perspectives and quantitative assessments | Narrative review | Low impact  Authors had noted It should be noted, however, that the evidence regarding pharmacological interventions is rather insufficient. |
| Acute Pharmacological Management of Behavioural and  Emotional Dysregulation following a Traumatic Brain Injury: A  Systematic Review of the Literature | Systematic review | Low to Moderate quality paper cited as limited number of high-quality studies, including only 5 RCT and more observational studies |
| Pharmacological Treatment of Agitation and/or Aggression in Patients With  Traumatic Brain Injury: A Systematic Review of Reviews | Literature review  11 systematic reviews included | Low to moderate quality  Data searches were limited to the past ten years and were not registered on a review protocol |
| Use of olanzapine to treat agitation in traumatic brain injury: a series of N of One trial | RCT | Moderate to high quality  3-5 participants only |
| Physicians’ beliefs and perceived importance of traumatic brain injury-associated agitation in critically ill patients: a survey of Canadian intensivists | 42 item survey for clinicians in ICU | Low quality  Self-administered survey  Authors declare a limited number of physicians working in level 1 trauma care centre |
| Updated Canadian Clinical Practice  Guideline for the Rehabilitation of  Adults With Moderate to Severe  Traumatic Brain Injury: Behavioural Recommendations | Systematic search of evidence-based review | Expert panels reviewed and discussed the new and existing evidence, evaluated its quality, and added, removed, or modified recommendations and tools as needed  Moderate quality |

Final Reason for inclusion/exclusion

1)Inclusion …………………………………………….

2) Exclusion

a) Does not satisfy Inclusion criteria ……………….

b) Ticks an exclusion criterion………………………….

CASP checklist

1) Was there a clear statement of the aims of the research Y/N/can’t tell (CT)

2) Is the qualitative methodology appropriate Y/N/can’t tell (CT)

3) Was the research design appropriate to address the aims of the research Y/N/can’t tell (CT)

4) Was the recruitment strategy appropriate to the aims of the research Y/N/can’t tell (CT)

5) Was the data collected in a way that addressed the research issue Y/N/can’t tell (CT)

6) Has the relationship between researcher and participants been adequately considered? Y/N/can’t tell (CT)

7) Have ethical issues been taken into consideration? Y/N/can’t tell (CT)

8) Was the data analysis sufficiently rigorous? Y/N/can’t tell (CT)

9) Is there a clear statement of findings? Y/N/can’t tell (CT)

10) How valuable is the research? Y/N/can’t tell (CT)

**Outcome:**

**Positive/methodology sound**

**Negative/relatively poor methodology**

**Unknown**

**Supplementary table 5 matrix detailing the breakdown of the CASP scores for each of the included studies in the review**

| **study** | **1** | **2** | **3** | **4** | **5** | **6** | **7** | **8** | **9** | **10** |
| --- | --- | --- | --- | --- | --- | --- | --- | --- | --- | --- |
| **1** | **Y** | **Y** | **Y** | **Y** | **CT** | **Y** | **Y** | **y** | **y** | **y** |
| **2** | **Y** | **Y** | **Y** | **CT** | **Y** | **CT** | **Y** | **n** | **y** | **n** |
| **3** | **Y** | **Y** | **Y** | **CT** | **Y** | **CT** | **Y** | **y** | **y** | **y** |
| **4** | **y** | **CT** | **y** | **CT** | **N/A prospective study** | **CT** | **Y** | **N/A prospective study** | **N/A prospective study** | **n** |
| **5** | **Drug trial**  **N/A** | **Drug trial**  **N/A** | **Drug trial**  **N/A** | **Drug trial**  **N/A** | **Drug trial**  **N/A** | **Drug trial**  **N/A** | **Drug trial**  **N/A** | **Drug trial**  **N/A** | **Drug trial**  **N/A** | **Drug trial**  **N/A** |
| **6** | **Y** | **Y** | **y** | **Y** | **Y** | **CT** | **Y** | **CN** | **y** | **n** |
| **7** | **Drug trial**  **N/A** | **Drug trial**  **N/A** | **Drug trial**  **N/A** | **Drug trial**  **N/A** | **Drug trial**  **N/A** | **Drug trial**  **N/A** | **Drug trial**  **N/A** | **Drug trial**  **N/A** | **Drug trial**  **N/A** | **Drug trial**  **N/A** |
| **8** | **Y** | **y** | **Y** | **CT** | **Y** | **CT** | **Y** | **n** | **y** | **n** |
| **9** | **y** | **y** | **Y** | **CT** | **Y** | **CT** | **Y** | **y** | **y** | **y** |
| **10** | **y** | **y** | **Y** | **CT** | **Y** | **CT** | **Y** | **y** | **y** | **n** |
| **11** | **N/A RCT** | **N/A RCT** | **N/A RCT** | **N/A RCT** | **N/A RCT** | **N/A RCT** | **N/A RCT** | **N/A RCT** | **N/A RCT** | **N/A RCT** |
| **12** | **y** | **y** | **Y** | **Y** | **Y** | **y** | **y** | **y** | **y** | **n** |
| **13** | **y** | **y** | **Y** | **CT** | **Y** | **CT** | **y** | **y** | **y** | **y** |

**Supplementary table 6 Outcome from the CASP checklist**

| **study** | **Outcome** |
| --- | --- |
| 1)Managing agitation during early recovery in adults with traumatic  brain injury: An international survey | **Positive/methodology sound** |
| 2)Effectiveness of Non‐Pharmacological Interventions for Agitation  during Post‐Traumatic Amnesia following Traumatic Brain Injury:  A Systematic Review | **Negative/relatively poor methodology** |
| 3)Pharmacological interventions for agitation  in patients with traumatic brain injury:  protocol for a systematic review and meta-  analysis | **Positive/methodology sound** |
| 4)Experiences and attitudes towards  Agitated behaviours in Traumatic brain  injury in the Intensive Care unit  patients (EXSTATIC): a protocol for an  interprofessional mixed-method study | **Unknown**  **Prospective trial CASP N/A** |
| 5)Safety of dexmedetomidine for the control of agitation in  critically ill traumatic brain injury patients: a descriptive study | **Unknown**  **Drug trial CASP N/A** |
| 6)Disrupted sleep predicts next day agitation  following moderate to severe brain injury | Low impact due to observational study  **Mixed outcome unknown** |
| 7)A descriptive analysis of pharmacological management of aggression and/or agitation in patients with traumatic brain injury in a Southwest Virginia inpatient population | **Unknown**  **Drug trial CASP N/A** |
| 8)Enhancing management of agitation after traumatic brain injury: Psychiatric perspectives and quantitative assessments | **Negative/relatively poor methodology** |
| 9)Acute Pharmacological Management of Behavioural and  Emotional Dysregulation following a Traumatic Brain Injury: A  Systematic Review of the Literature | **Positive/methodology sound** |
| 10)Pharmacological Treatment of Agitation and/or Aggression in Patients With  Traumatic Brain Injury: A Systematic Review of Reviews | **Positive/methodology sound** |
| 11)Use of olanzapine to treat agitation in traumatic brain injury: a series of N of One trial | **Unknown**  **RCT – CASP N/A** |
| 12)Physicians’ beliefs and perceived importance of traumatic brain injury-associated agitation in critically ill patients: a survey of Canadian intensivists | **Negative/relatively poor methodology** |
| 13)Updated Canadian Clinical Practice  Guideline for the Rehabilitation of  Adults With Moderate to Severe  Traumatic Brain Injury: Behavioural Recommendations | **Positive/methodology sound** |

Appendices: Prisma checklist (2020)

**Topic**

**Item**

**# Checklist item TITLE**

Title 1 Identify the report as a scoping review.

**ABSTRACT**

Title 1 Identify the report as a scoping review. Response Completed

**BACKGROUND**

Objectives 2 Provide an explicit statement of the main objective(s) or question(s) the review addresses.

Response Completed

**METHODS**

Eligibility criteria

3 Specify the inclusion and exclusion criteria for the review.

Information sources

Response Completed

4 Specify the information sources (e.g. databases, registers) used to identify studies and the date when each.

Was last searched.

Response Completed

Risk of bias

5 Specify the methods used to assess risk of bias in the included studies.

Synthesis of results

Response Completed, CASP checklist used.

6 Specify the methods used to present and synthesise results.

None to declare.

**RESULTS**

Included studies.

7 Give the total number of included studies and participants and summarise relevant characteristics of studies.

Synthesis of results -completed.

8 Present results for main outcomes, preferably indicating the number of included studies and participants for

each. If meta-analysis was done, report the summary estimate and confidence/credible interval. If comparing

groups, indicate the direction of the effect (i.e. which group is favoured).

Response Completed

**DISCUSSION**

Limitations of evidence

9 Provide a summary of the limitations of the evidence included in the review (e.g. study risk of bias,

inconsistency and imprecision)- point made in the discussion.

Interpretation

10 Provide a general interpretation of the results and important implications-

Response Completed

**OTHER**

Funding

11 Specify the primary source of funding for the review. - no funding to declare

Registration

12 Provide the register name and registration number. - Prospero provided

**INTRODUCTION**

Rationale 3 Describe the rationale for the review in the context of existing knowledge. Objectives 4 provide an explicit statement of the objective(s) or question(s) the review addresses.

Response Completed

**METHODS**

Eligibility criteria

5 Specify the inclusion and exclusion criteria for the review and how studies were grouped for the syntheses. Information

Sources

6 Specify all databases, registers, websites, organisations, reference lists and other sources searched or consulted to identify studies.

Specify the date when each source was last searched or consulted.

Search strategy.

Response Completed

7 Present the full search strategies for all databases, registers and websites, including any filters and limits used.

Response Completed

8 Specify the methods used to decide whether a study met the inclusion criteria of the review, including how many reviewers screened each.

record and each report retrieved, whether they worked independently, and if applicable, details of automation tools used in the process. Data collection Process

Response Completed

9 Specify the methods used to collect data from reports, including how many reviewers collected data from each report, whether they worked independently, any processes for obtaining or confirming data from study investigators, and if applicable, details of automation tools used in the process.

Response Completed

Data items

10a List and define all outcomes for which data were sought. Specify whether all results that were compatible with each outcome domain in each study were sought (e.g. for all measures, time points, analyses), and if not, the methods used to decide which results to collect.

Response Completed

10b List and define all other variables for which data were sought (e.g. participant and intervention characteristics, funding sources). Describe any assumptions made about any missing or unclear information. It was noted in the results section that one study had a lack of reliability as data for the end point of agitation was not collected for all patients receiving a drug.

Study risk of bias Assessment

11 Specify the methods used to assess risk of bias in the included studies, including details of the tool(s) used, how many reviewers assessed. Each study and whether they worked independently, and if applicable, details of automation tools used in the process.

Effect measures.

Response Completed

12 Specify for each outcome the effect measure(s) (e.g. risk ratio, mean difference) used in the synthesis or presentation of results. Synthesis

Methods

Response Completed

13a Describe the processes used to decide which studies were eligible for each synthesis (e.g. tabulating the study intervention characteristics and comparing against the planned groups for each synthesis (item #5).

Response Completed

13b Describe any methods required to prepare the data for presentation or synthesis, such as handling of missing summary statistics, or data conversions.

Response Completed

13c Describe any methods used to tabulate or visually display results of individual studies and syntheses. **L**

Response Completed

**Item**

13d Describe any methods used to synthesize results and provide a rationale for the choice(s). If meta-analysis was performed, describe the model(s), method(s) to identify the presence and extent of statistical heterogeneity, and software package(s) used.

Response Completed

13e Describe any methods used to explore possible causes of heterogeneity among study results (e.g. subgroup analysis, meta-regression). None needed.

13f Describe any sensitivity analyses conducted to assess robustness of the synthesized results. Reporting bias

Assessment JBI and CASP assessment used for bias.

14 Describe any methods used to assess risk of bias due to missing results in a synthesis (arising from reporting biases). Certainty

JBI and CASP assessment used for bias.

15 Describe any methods used to assess certainty (or confidence) in the body of evidence for an outcome.

None used.

**RESULTS**

Study selection

16a Describe the results of the search and selection process, from the number of records identified in the search to the number of studies. Included in the review, ideally using a flow diagram.

Response Completed

16b Cite studies that might appear to meet the inclusion criteria, but which were excluded, and explain why they were excluded. Study Characteristics

Response Completed

17 Cite each included study and present its characteristics. Risk of bias in Studies

Response Completed

18 Present assessments of risk of bias for each included study. Results of individual studies

Response Completed in results section noting the limitations of the studies.

19 For all outcomes, present, for each study: (a) summary statistics for each group (where appropriate) and (b) an effect estimates and its precision (e.g. confidence/credible interval), ideally using structured tables or plots.

Not added for grouped data but for individual studies

Results of syntheses

20a for each synthesis, briefly summarise the characteristics and risk of bias among contributing studies.

The results section has noted limitations of the results.

20b Present results of all statistical syntheses conducted. If meta-analysis was done, present for each the summary estimate and its precision. (e.g. confidence/credible interval) and measures of statistical heterogeneity. If comparing groups, describe the direction of the effect. Many of the studies included were from different topics after exclusion and not able to be compared necessarily by confidence intervals.

20c Present results of all investigations of possible causes of heterogeneity among study results.

Explanation of different results was seen in the method section.

20d Present results of all sensitivity analyses conducted to assess the robustness of the synthesized results. Reporting biases

CASP and JBI

21 Present assessments of risk of bias due to missing results (arising from reporting biases) for each synthesis assessed. Certainty of Evidence

Results section

22 Present assessments of certainty (or confidence) in the body of evidence for each outcome assessed.

None to declare.

**DISCUSSION**

23a Provide a general interpretation of the results in the context of other evidence. Response Completed

23b Discuss any limitations of the evidence included in the review. Response Completed

23c Discuss any limitations of the review processes used. Response Completed

23d Discuss implications of the results for practice, policy, and future research. Response Completed

**OTHER INFORMATION**

Registration and protocol

24a Provide registration information for the review, including register name and registration number, or state that the review was not registered. Methods, Registration and Protocol. Response Completed

24b Indicate where the review protocol can be accessed or state that a protocol was not prepared. Methods,

Registration and Protocol. Prospero- Response Completed

24c Describe and explain any amendments to information provided at registration or in the protocol. Methods,

Registration and Protocol Support No amendments.

25 Describe sources of financial or non-financial support for the review, and the role of the funders or sponsors in the review. Acknowledgments Competing interests. No conflicts and no funding source

26 Declare any competing interests of review authors. Declaration of

Conflicting Interests Availability of data, code and other materials No conflicts and no funding source, nothing else to declare.

27 Report which of the following are publicly available and where they can be found: template data collection forms; data extracted from included studies; data used for all analyses; analytic code; any other materials used in the review.

Study registered on Prospero.

Data Availability

Statement

*From:* Page MJ, McKenzie JE, Bossu
